# Six Versus Twelve Months Clopidogrel Therapy After Drug-Eluting Stenting in Patients With Acute Coronary Syndrome: An ISAR-SAFE Study Subgroup Analysis

**DOI:** 10.1038/srep33054

**Published:** 2016-09-14

**Authors:** Raphaela Lohaus, Jonathan Michel, Katharina Mayer, Anna Lena Lahmann, Robert A. Byrne, Annabelle Wolk, Jurrien M. ten Berg, Franz-Josef Neumann, Yaling Han, Tom Adriaenssens, Ralph Tölg, Melchior Seyfarth, Michael Maeng, Bernhard Zrenner, Claudius Jacobshagen, Jochen Wöhrle, Sebastian Kufner, Tanja Morath, Tareq Ibrahim, Isabell Bernlochner, Marcus Fischer, Heribert Schunkert, Karl-Ludwig Laugwitz, Julinda Mehilli, Adnan Kastrati, Stefanie Schulz-Schüpke

**Affiliations:** 1 Deutsches Herzzentrum München, Technische Universität, Munich, Germany; 2 Universitätsklinikum Goettingen, Goettingen, Germany; 3 St. Antonius Hospital, Nieuwegein, Netherlands; 4 Universitäts-Herzzentrum Freiburg – Bad Krozingen, Bad Krozingen, Germany; 5 Shenyang Northern Hosp, Shenyang, China; 6 University Hospital Leuven, Leuven, Belgium; 7 Herzzentrum der Segeberger Kliniken Gruppe, Bad Segeberg, Germany; 8 Helios Klinik Wuppertal, Wuppertal, Germany; 9 Aarhus University Hospital, Aarhus, Denmark; 10 Krankenhaus Landshut-Achdorf, Landshut, Germany; 11 Universitätsklinikum Ulm, Ulm, Germany; 12 1. Medizinische Klinik, Klinikum rechts der Isar, Technische Universität, Munich, Germany; 13 Universitätsklinikum Regensburg, Regensburg, Germany; 14 DZHK, Partner Site Munich Heart Alliance, Munich, Germany; 15 Munich University Clinic, Ludwig-Maximilians University, Munich, Germany

## Abstract

In patients presenting with acute coronary syndrome (ACS) the optimal duration of dual-antiplatelet therapy after drug-eluting stent (DES) implantation remains unclear. At 6 months after intervention, patients receiving clopidogrel were randomly assigned to either a further 6-month period of placebo or clopidogrel. The primary composite endpoint was death, myocardial infarction, stent thrombosis, stroke, or major bleeding 9 months after randomization. The ISAR-SAFE trial was terminated early due to low event rates and slow recruitment. 1601/4000 (40.0%) patients presented with ACS and were randomized to 6 (n = 794) or 12 months (n = 807) clopidogrel. The primary endpoint occurred in 14 patients (1.8%) receiving 6 months of clopidogrel and 17 patients (2.2%) receiving 12 months; hazard ratio (HR) 0.83, 95% confidence interval (CI) 0.41–1.68, P = 0.60. There were 2 (0.3%) cases of stent thrombosis in each group; HR 1.00, 95% CI 0.14–7.09, P = >0.99. Major bleeding occurred in 3 patients (0.4%) receiving 6 months clopidogrel and 5 (0.6%) receiving 12 months; HR 0.60, 95% CI 0.15–2.49, P = 0.49. There was no significant difference in net clinical outcomes after DES implantation in ACS patients treated with 6 versus 12 months clopidogrel. Ischaemic and bleeding events were low beyond 6-months.

Patients presenting with acute coronary syndrome (ACS) are at higher risk of subsequent ischaemic events compared to those with stable angina. Dual antiplatelet therapy (DAPT) with acetylsalicylic acid and an adenosine diphosphate receptor antagonist reduces ischaemic events in this population – although increases bleeding – regardless of whether an invasive or medical treatment strategy is chosen^1^,^2^. Improvements in stent design, delivery technique, and the widespread adoption of DAPT have reduced the rate of stent thrombosis after PCI^3^.

Although prolonged DAPT reduces ischaemic endpoints after intervention the increased risk of bleeding may limit the clinical efficacy and the optimal duration of DAPT after percutaneous coronary intervention (PCI) in patients presenting with ACS remains controversial. U.S. and European guidelines currently recommend 12 months of DAPT for ACS patients, without an indication for oral anticoagulation, undergoing PCI; this recommendation is based on previous observational and registry data raising concern about the ongoing risk of stent thrombosis with early generation DES^4^,^5^ and evidence of incremental benefit over 12 months demonstrated in the CURE trial^1^. With respect to the treatment effect of shorter duration (<12 months) DAPT after PCI, randomised trials have been limited by methodological difficulties, including inadequate sample size^6^,^7^,^8^,^9^,^10^,^11^, however, meta-analyses of available data have shown lower rates of bleeding with no increase in ischaemic events in those treated with shorter duration DAPT^12^,^13^.

The ISAR-SAFE study was designed to assess 6 versus 12 months clopidogrel for patients undergoing DES implantation for both stable coronary disease and ACS^11^. The trial demonstrated non-inferiority of the 6-month clopidogrel regimen in terms of net clinical outcome – the composite of death, myocardial infarction, stent thrombosis, stroke and major bleeding at 9 months after randomization (15 months after DES implantation). The study findings were limited by lower than expected event rates and early termination due to slow enrolment. This ISAR-SAFE ACS report is a prespecified subgroup analysis that aims to examine the treatment effect of 6 versus 12 months clopidogrel for ACS patients enrolled in the ISAR-SAFE trial.

## Methods

The current report is a prespecified subgroup analysis of the ACS patients recruited for the ISAR-SAFE trial. Full details of the ISAR-SAFE trial design have previously been published^11^,^14^. ISAR-SAFE was an investigator-initiated, international, multicenter, randomised, double-blind, placebo-controlled, non-inferiority trial that sought to assess 6 months versus 12 months duration of clopidogrel treatment for patients undergoing DES implantation. Patients were eligible for enrolment if they were receiving clopidogrel therapy at 6 (−1/+2) months after DES implantation and had provided written informed consent. Major exclusion criteria were age ≤ 18 years, clinical symptoms or proof of ischemia and/or angiographic lesions requiring revascularization, previous stent thrombosis, DES implantation in the left main coronary artery, myocardial infarction during the 6 months after DES implantation, malignancies or other comorbid conditions with a life expectancy of <1 year or that may result in protocol noncompliance, planned major surgery within 6 months with the need to discontinue antiplatelet therapy, active bleeding, bleeding diathesis, history of intracranial bleeding, oral anticoagulation, and known allergy or intolerance of the study medication. Enrolled patients were subsequently randomised 1:1 to receive either placebo or clopidogrel for 6 months, in addition to acetyl salicylic acid, and other medications prescribed at the discretion of the treating physician. All patients were scheduled to undergo clinical follow-up at 30 days, 6 months and 9 months after randomization. The primary endpoint was the composite of death, myocardial infarction, stent thrombosis (definite or probable), stroke, or Thrombolysis in Myocardial Infarction (TIMI) major bleeding at 9 months after randomization, i.e. 15 months after the index intervention. Secondary endpoints were the individual components of the primary endpoint. The study was conducted in accordance with the Declaration of Helsinki. The Ethics Committee of the Technische Universität München (Ismaninger Straße 22, 81675 München, Germany), and each participating center, approved the study. All patients provided written informed consent.

### Statistical Considerations

The statistical design of the non-inferiority ISAR-SAFE study has been described in detail previously^11^,^14^. Categorical variables were summarized using frequencies and proportions and compared using the chi-square test or Fisher’s exact test, as appropriate. Continuous data were summarized using mean ± standard deviation or median [interquartile range] and compared using Student’s t-test or nonparametric Wilcoxon rank-sum test, respectively. Cumulative event rates were calculated by the Kaplan Meier method and a Cox proportional hazards model was used for the comparison of the 2 study groups. The primary end point was also assessed in subgroups defined by age, gender, presence of diabetes mellitus, previous myocardial infarction, complexity of the treated lesion (complex lesions defined as B2 and C per the modified American College of Cardiology/American Heart Association Task Force classification), impaired left ventricular function (<55%), early generation DES, and premature study drug discontinuation. Software R (version 2.15.2, The R Foundation for Statistical Computing) was used for statistical analyses. The significance level was set at a two-sided alpha level of 0.05.

## Results

### Patients and Procedures

Between September 2006 and April 2014 a total of 1601/4000 (40.0%) patients enrolled in the ISAR-SAFE trial presented with ACS: 794 were randomly assigned to receive 6 months of clopidogrel and 807 to 12 months.

Table 1 summarizes the baseline clinical and demographic characteristics according to treatment allocation to 6 or 12 months of clopidogrel. Angiographic and procedural characteristics at the time of index intervention are shown in Table 2. Overall 734/1601 (45.8%) patients presented with myocardial infarction at the index intervention: 410 patients (25.6%) with an acute non-ST elevation myocardial infarction and 324 (20.2%) with ST elevation myocardial infarction. Table 3 shows the medication patients were receiving at the time of randomization.

Premature discontinuation of the study drug was recorded in 12.0% of the patients assigned to 6 months of clopidogrel and 14.7% of the patients assigned to 12 months of clopidogrel (p = 0.10).

### Clinical Outcomes

There was no signal for interaction to suggest an overall treatment effect in this ACS population (P_interaction_ = 0.72). Clinical follow-up at 9 months was available for all but 84 patients, 34 assigned to 6 months of clopidogrel (4.3%) and 50 patients (6.3%) assigned to 12 months; P = 0.08. In patients with incomplete 9-month follow-up, the median duration of follow-up was 190 [IQR 180–226] days. A total of 21 patients (1.4%), 8 patients assigned to 6 months of clopidogrel and 13 patients assigned to 12 months of clopidogrel, had a follow-up shorter than 6 months (P = 0.28).

**Figure 1:**
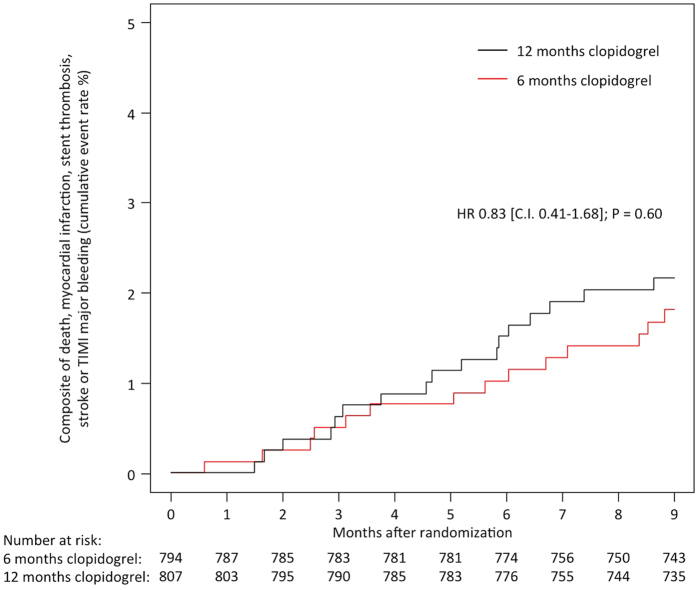
Primary Composite Endpoint of Death, Myocardial Infarction, Stent Thrombosis, Stroke or TIMI Major Bleeding at 9 Months in the Two Study Groups of Six and Twelve Months Clopidogrel Therapy.

Clinical outcomes according to study group are reported in Table 4. At 9 months, the composite primary endpoint of death, myocardial infarction (MI), stent thrombosis, stroke or major bleeding was observed in 14 patients (1.8%) assigned to 6 months of clopidogrel and 17 patients (2.2%) assigned to 12 months; hazard ratio (HR) 0.83, 95% confidence interval (CI) 0.41–1.68, P = 0.60 (graphical representation of primary endpoint shown in Fig. 1).

**Figure 2:**
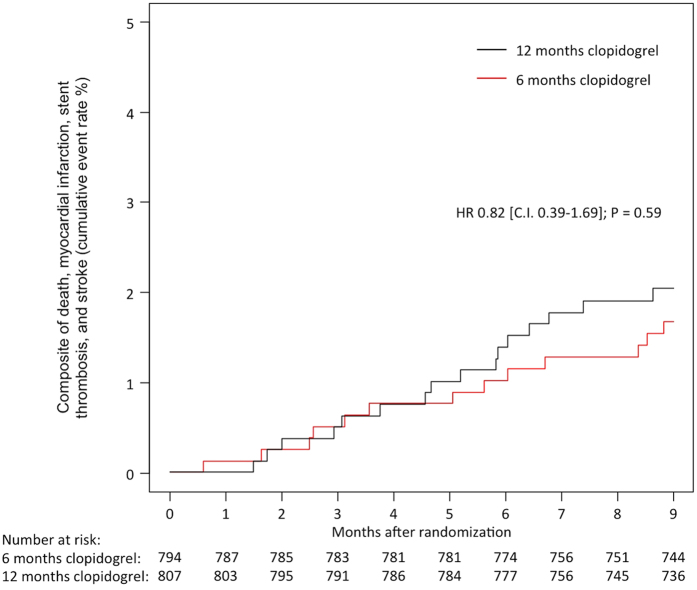
Composite of Death, Myocardial Infarction, Stent Thrombosis or Stroke at 9 Months in the Two Study Groups of Six and Twelve Months Clopidogrel Therapy.

Regarding the secondary endpoints, 5 patients (0.6%) assigned to 6 months of clopidogrel and 7 patients (0.9%) assigned to 12 months of clopidogrel died within 9 months; HR 0.72, 95% CI 0.23–2.26, P = 0.57. There were 4 cases of definite stent thrombosis, 2 (0.3%) in patients assigned to 6 months of clopidogrel and 2 (0.3%) in patients assigned to 12 months of clopidogrel; HR 1.00, 95% CI 0.14–7.09, P = >0.99. The rates of MI and stroke were low and comparable in both study groups (MI: 6 patients [0.8%] vs. 8 patients (1.0%); HR 0.75, 95% CI 0.26–2.17, P = 0.60 and stroke: 3 patients [0.4%] vs. 2 [0.3%]; HR 0.75, 95% CI 0.26–2.17, P = 0.65, in the 6 month and 12 month groups respectively). The composite of death, MI, stent thrombosis, or stroke is shown in Fig. 2.

**Figure 3:**
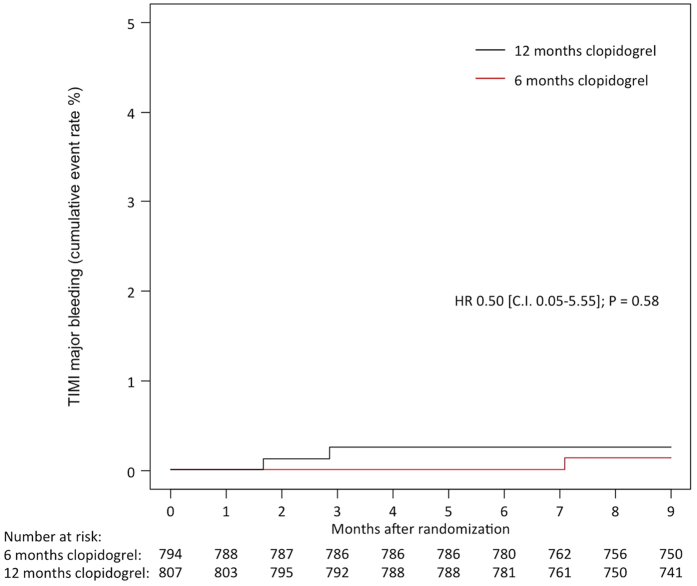
TIMI Major Bleeding at 9 Months in the Two Study Groups of Six and Twelve Months Clopidogrel Therapy.

**Figure 4:**
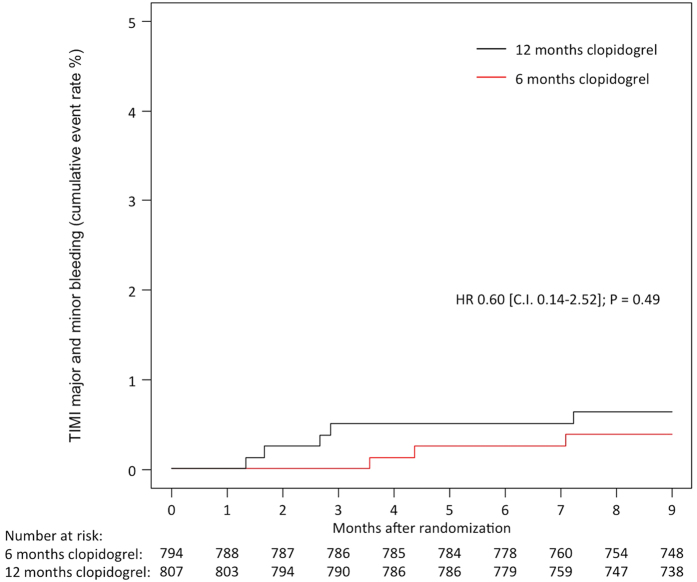
Composite of TIMI Major or Minor Bleeding at 9 Months in the Two Study Groups of Six and Twelve Months Clopidogrel Therapy.

TIMI major bleeding was observed in 1 patient (0.1%) assigned to 6 months of clopidogrel and in 2 patients (0.3%) assigned to 12 months of clopidogrel; HR 0.50, 95% CI 0.05–5.55, P = 0.58 (Fig. 3). There was no significant reduction in the rate of TIMI minor bleeding in patients receiving 6 months of clopidogrel (2 events [0.3%] vs. 3 events [0.4%]; HR 0.67, 95% CI 0.11–3.96, P = 0.66), nor the composite of TIMI major and minor bleeding (3 events [0.4%] versus 5 events; HR 0.60, 95% CI 0.14–2.52, P = 0.49) (Fig. 4). Bleeding, according to the Bleeding Academic Research Consortium (BARC), ≥ class 2 was observed in 9 patients (1.1%) assigned to 6 months clopidogrel and 17 patients (2.1%) assigned to 12 months clopidogrel (p = 0.12).

**Figure 5:**
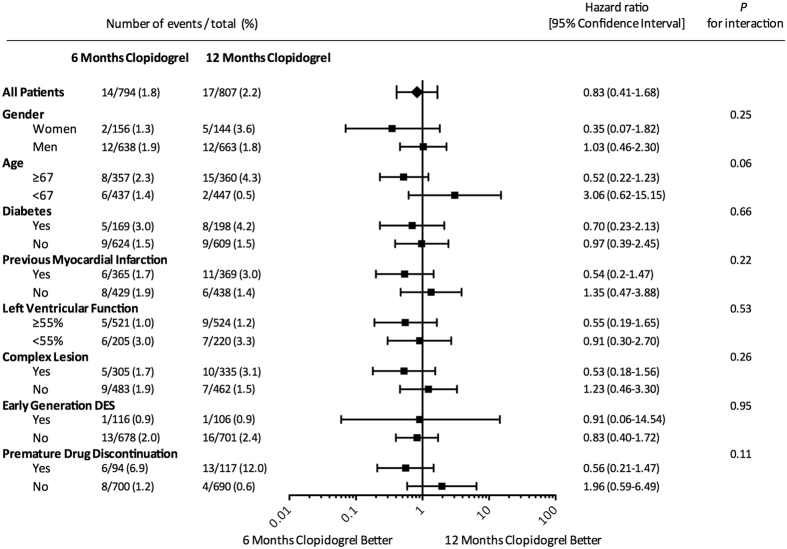
Incidence of The Primary Endpoint in Subgroup Analysis – DES = Drug Eluting Stent.

Results of subgroup analysis are shown in Fig. 5. There was no significant interaction regarding the primary endpoint between treatment effect and age (P_interaction_ = 0.06), diabetic versus non-diabetic patients (P_interaction_ = 0.66), previous MI versus no previous MI (P_interaction_ = 0.22), normal versus impaired LV function <55% (P_interaction_ = 0.53), simple versus complex lesion morphology (ACC/AHA classification B2 or C) (P_interaction_ = 0.26), early versus new generation DES (P_interaction_ = 0.95), and premature discontinuation of study drug (P_interaction_ = 0.11).

## Discussion

This prespecified subgroup analysis of the ISAR-SAFE study assessed the treatment effect of 6 months versus 12 months clopidogrel for patients presenting with ACS and receiving treatment with a DES. The main finding is that event rates of the primary composite end point were low and were not statistically significant between the two groups. This study was investigator-initiated, industry-independent, and to date is the only double-blind, placebo-controlled trial that randomised ACS patients to shorter versus standard duration DAPT after DES implantation.

Current guideline recommendations regarding the duration of DAPT after DES implantation were drawn from first-generation DES observational data that indicated a small ongoing risk of ischaemic events and stent thrombosis beyond 12 months^4^,^15^. Subsequent evolutions in DES drug and polymer coatings reduced the risk of late stent thrombosis in 2nd generation devices^16^, ultimately raising the question of whether early cessation of DAPT following 2nd generation DES implantation could improve overall clinical outcomes by reducing bleeding complications. Observational data identified ACS at presentation as an independent risk factor for stent thrombosis^4^ in comparison to those with stable coronary disease, and this is reflected in the guidelines by a longer recommended period of DAPT for ACS patients undergoing stent implantation^17^,^18^. However, ACS patients have frequently been underrepresented in subsequent trials exploring duration of DAPT after stent implantation, often as a result of inherent methodological difficulties, and the appropriate treatment strategy remains unclear.

Prior to our study, a number of randomised trials explored <12 months versus ≥12 months duration of DAPT after stent implantation. Several of these trials excluded, or restricted the inclusion, of high-risk patients^6^,^8^,^9^,^10^,^19^. Of the studies to include high-risk patients, this population has been underrepresented in some cases^20^, and the results from the largest available randomised trial are limited by underpowering for the primary endpoint, and an open-label design^7^.

Prolongation of DAPT therapy beyond the guideline recommended 12-month period may be an alternative strategy for ACS patients at low bleeding risk. For example, the ‘DAPT’ trial found lower ischaemic events with 30 months, compared to 12 months, of clopidogrel after DES at a cost of increased bleeding^21^. Recent meta-analyses including randomised trials of longer versus standard duration DAPT also support the notion that ischaemic endpoints and cardiovascular, but not overall, mortality are improved by ongoing therapy: counter-balanced by increased non-fatal major bleeding^22^,^23^.

In our double-blind, placebo-controlled study we included ACS patients, who remained stable six months after index PCI. The primary endpoint was a composite of ischaemic and bleeding events, which seeks to reflect net clinical benefit to the patient. Although conclusions from subgroup analyses must be carefully drawn, the main finding of low event rates and similar net clinical outcomes in ACS patients receiving clopidogrel for 6 months, compared with 12 months, lends some support to the hypothesis that a shorter duration of DAPT may also be a suitable management strategy for selected patients. Although this study is not powered to interpret further subanalysis, no heterogeneity of treatment effect was seen amongst other ACS population subgroups.

The ISAR-SAFE ACS substudy has a number of limitations that should be recognized. Firstly, patients in the ISAR-SAFE study were randomised 6 months after index PCI; as a result, there was an unavoidable bias toward enrolment of patients at lower ischaemic and bleeding risk and the findings cannot be generalized to the ACS population as a whole. Additionally, the requirement for patients to present to hospital 6 months after stent implantation, for eligibility assessment, is likely to introduce an element of bias in favour of enrolment of lower-risk ACS patients. Secondly, referring physicians may have preferred not to refer higher-risk ACS patients for consideration of enrolment given the potential for randomisation to shorter-term DAPT and a perceived increased risk of adverse events. Thirdly, although statistical power calculations were derived from historical trials using first-generation DES, 89% of patients enrolled in ISAR-SAFE received second-generation stents; these stents are associated with a lower rate of stent thrombosis and other complications compared with first-generation DES, leading to a reduction in ischaemic endpoints. Finally, novel P2Y12 inhibitors have emerged since the beginning of enrolment for the ISAR-SAFE study. ACS patients treated with ticagrelor have lower rates of ischaemic events compared to those receiving clopidogrel^24^ and patients with a previous history of myocardial infarction benefit from prolonged treatment with ticagrelor^25^. Given their proven superiority to clopidogrel, current guidelines recommend novel P2Y12 inhibitors as the preferred second antiplatelet agent for patients presenting with ACS^17^,^18^ and patients treated with these agents would not have been eligible for inclusion in ISAR-SAFE. Therefore, although this study includes the largest cohort of ACS patients randomised to shorter versus standard duration DAPT after DES implantation, and event rates are comparable to similarly designed trials^10^, we acknowledge the underpowering of the trial in relation to detection of differences in clinical outcomes. However, the data presented here remain relevant for inclusion in future meta-analyses exploring shorter versus standard duration of DAPT after DES.

In summary, whilst this study was underpowered for the primary endpoint, the ISAR-SAFE ACS subgroup analysis demonstrated low event rates and no statistically significant difference in net clinical outcomes, defined as death, myocardial infarction, stroke, or TIMI major bleeding, for patients presenting with ACS receiving 6 months versus 12 months clopidogrel after DES implantation.

## Additional Information

**How to cite this article**: Lohaus, R. *et al*. Six Versus Twelve Months Clopidogrel Therapy After Drug-Eluting Stenting in Patients With Acute Coronary Syndrome: An ISAR-SAFE Study Subgroup Analysis. *Sci. Rep*. **6**, 33054; doi: 10.1038/srep33054 (2016).

## Supplementary Material

Supplementary Information

## Figures and Tables

**Table 1:** Baseline Clinical and Demographic Characteristics at the Time of Randomization.

	Six Months Clopidogrel (n = 794)	Twelve Months Clopidogrel (n = 807)	*P*
Age, years	64.8 [57.4–72.8]	64.7 [56.4–73.0]	0.77
Female	156/794 (19.6)	144/807 (17.8)	0.36
Arterial hypertension	685/791 (86.6)	719/807 (89.1)	0.13
Hypercholesterolemia	646/793 (81.5)	666/807 (85.5)	0.58
Diabetes mellitus	169/793 (21.3)	198/807 (24.5)	0.13
Insulin-requiring	53/793 (6.7)	70/807 (8.7)	0.14
Family history	253/755 (33.5)	246/763 (32.2)	0.60
Smoking status			
Active smoker	141/793 (17.8)	136/807 (16.9)	0.62
Former smoker	273/793 (34.4)	291/807 (36.1)	0.49
History of prior myocardial infarction	181/792 (22.9)	178/807 (22.1)	0.70
History of prior coronary artery bypass graft	47/787 (6.0)	59/798 (7.4)	0.26
Body mass index, kg/m^2^	27.6 [24.7–29.6]	27.7 [24.7–30.3]	0.74

Data are shown as number (percentage) or median [interquartile range].

**Table 2:** Angiographic and Procedural Characteristics at the Time of Index Intervention.

	Six Months Clopidogrel (n = 794)	Twelve Months Clopidogrel (n = 807)	*P*
Clinical presentation			0.89
Unstable angina	429/794 (54.0)	438/807 (54.3)	
NSTEMI	207/794 (26.1)	203/807 (25.2)	
STEMI	158/794 (19.9)	166/807 (20.6)	
Reduced left ventricular- ejection fraction (<55%)	205/726 (28.2)	220/744 (29.6)	0.57
Number of diseased vessels			0.41
1	364/794 (45.8)	345/807 (42.8)	
2	237/794 (29.8)	262/807 (32.5)	
3	193/794 (24.3)	200/807 (24.8)	
Multivessel disease	430/794 (54.2)	462/807 (57.2)	0.21
Target vessel			0.57
LAD	329/794 (41.1)	323/807 (40.0)	
LCx	205/794 (25.8)	194/807 (24.0)	
RCA	245/794 (30.9)	279/807 (34.6)	
LMCA	3/794 (0.4)	2/807 (0.2)	
Bypass graft	12/794 (1.5)	9/807 (1.1)	
Lesion characteristics			
Complex lesion	305/788 (38.7)	335/797 (42.0)	0.18
Chronic total occlusion	62/794 (7.8)	48/804 (6.0)	0.15
Bifurcation lesion	134/794 (16.9)	139/804 (17.3)	0.83
Vessel size, mm	3.0 [2.75–3.5]	3.0 [2.75–3.5]	0.48
Multilesion intervention	284/794 (35.8)	298/807 (36.9)	0.63
Drug-eluting stent type			0.18
PES	21/794 (2.6)	17/807 (2.1)	
Early generation SES	95/794 (12.0)	89/807 (11.0)	
New generation SES	126/794 (15.9)	134/807 (16.6)	
EES	368/794 (46.3)	402/807 (49.8)	
ZES	123/794 (15.5)	95/807 (11.8)	
BES	56/794 (7.1)	63/807 (7.8)	
BVS	1/794 (0.1)	0/807 (0.0)	
BMS	4/794 (0.5)	3/807 (0.4)	
Drug-coated balloon	0/794 (0.4)	4/807 (0.5)	
POBA	0/794 (0.0)	0/807 (0.0)	
Number of stents	1.64 ± 0.94	1.68 ± 0.93	0.36
Total stented length, mm	28 [18–44]	28 [18–45.75]	0.36

Data are shown as number (percentage), mean ± standard deviation or median [interquartile range]. NSTEMI  =  Non-ST segment elevation myocardial infarction, STEMI  =  ST elevation myocardial infarction, LAD  =  left anterior descending, LCx  =  left circumflex, RCA  =  right coronary artery, LMCA  =  left main coronary artery, PES  =  paclitaxel-eluting stent, SES  =  sirolimus-eluting stent, EES  =  everolimus-eluting stent, ZES  =  zotarolimus-eluting stent, BES  =  biolimus-eluting stent, BVS  =  bioresorbable everolimus-eluting vascular scaffold, POBA  =  plain balloon angioplasty.

**Table 3:** Medication at Randomisation.

	Six Months Clopidogrel (n = 794)	Twelve Months Clopidogrel (n = 807)	*P*
Acetylsalicylic acid	794/794 (100)	806/807 (99.9)†	0.32
Beta blocker	681/794 (85.8)	703/807 (87.1)	0.43
ACE inhibitor*	509/794 (64.1)	545/807 (67.5)	0.15
Angiotensin II receptor blocker	146/793 (18.4)	148/806 (18.4)	0.98
Calcium antagonist	148/793 (18.7)	152/807 (18.8)	0.93
Diuretic	233/792 (29.4)	227/807 (28.1)	0.57
Proton pump inhibitor	215/787 (27.3)	224/802 (27.9)	0.79
Statin	763/794 (96.1)	765/807 (94.8)	0.21

Data are shown as number (percentage).

^*^ACE = Angiotensin-Converting Enzyme.

^†^One patient in the twelve months clopidogrel group was treated with cilostazol instead of acetylsalicylic acid.

**Table 4:** Clinical Outcomes at 9 Months.

	Six Months Clopidogrel (n = 794)	Twelve Months Clopidogrel (n = 807)	HR (95% CI)	*P*
Primary endpoint — composite of death, myocardial infarction, definite or probable stent thrombosis, stroke or TIMI* major bleeding)	14 (1.8)	17 (2.2)	0.83 (0.41–1.68)	0.60
Secondary endpoints				
Death	5 (0.6)	7 (0.9)	0.72 (0.23–2.26)	0.57
Myocardial infarction	6 (0.8)	8 (1.0)	0.75 (0.26–2.18)	0.60
Definite or probable stent thrombosis	2 (0.3)	2 (0.3)	1.00 (0.14–7.09)	>0.99
Stroke	3 (0.4)	2 (0.3)	1.51 (0.25–9.01)	0.65
TIMI* major Bleeding	1 (0.1)	2 (0.3)	0.50 (0.05–5.55)	0.58
Composite of death, myocardial infarction, definite or probable stent thrombosis, or stroke)	13 (1.7)	16 (2.0)	0.82 (0.39–1.69)	0.59
Definite stent thrombosis	2 (0.3)	2 (0.3)	1.00 (0.14–7.09)	>0.99
TIMI* minor bleeding	2 (0.3)	3 (0.4)	0.67 (0.11–4.01)	0.66
TIMI* major or minor bleeding	3 (0.4)	5 (0.6)	0.60 (0.14–2.52)	0.49

Data are shown as number (percentage).

^*^TIMI  =  Thrombolysis in Myocardial Infarction.
