# Supplementary material for: Six Versus Twelve Months Clopidogrel Therapy After Drug-Eluting Stenting in Patients With Acute Coronary Syndrome: An ISAR-SAFE Study Subgroup Analysis
Source: Sci Rep. 2016 Sep 14;6:33054. doi: 10.1038/srep33054 (PMC5021963; doi:10.1038/srep33054)
Supplement: Supplementary Information [file srep33054-s1.pdf]

Appendix: ISAR-SAFE ACS Subgroup Analysis

Six Versus Twelve Months Clopidogrel Therapy After Drug-Eluting Stenting in Patients With Acute Coronary Syndrome: An ISAR-SAFE Study Subgroup Analysis

Authors: [§]Raphaela Lohaus[1], [§]*Jonathan Michel[1], Katharina Mayer[1], Anna Lena Lahmann[1], Robert A. Byrne[1], Annabelle Wolk[2], Jurrien M. ten Berg[3], Franz-Josef Neumann[4], Yaling Han[5], Tom Adriaenssens[6], Ralph Tölg[7], Melchior Seyfarth[8], Michael Maeng[9], Bernhard Zrenner[10], Claudius Jacobshagen[2], Jochen Wöhrle[11], Sebastian Kufner[1], Tanja Morath[1], Tareq Ibrahim[12], Isabell Bernlochner[12], Marcus Fischer[13], Heribert Schunkert[1,14], Karl-Ludwig Laugwitz[12,14], Julinda Mehilli[14,15], Adnan Kastrati[1,14], Stefanie Schulz-Schüpke [1,14]

From: [1] Deutsches Herzzentrum München, Technische Universität, Munich, Germany; [2] Universitätsklinikum Goettingen, Goettingen, Germany; [3] St. Antonius Hospital, Nieuwegein, Netherlands; [4] Universitäts-Herzzentrum Freiburg – Bad Krozingen, Bad Krozingen, Germany; [5] Shenyang Northern Hosp, Shenyang, China; [6] University Hospital Leuven, Leuven, Belgium; [7] Herzzentrum der Segeberger Kliniken Gruppe, Bad Segeberg, Germany; [8] Helios Klinik Wuppertal, Wuppertal, Germany; [9] Aarhus University Hospital, Aarhus, Denmark; [10] Krankenhaus Landshut-Achdorf, Landshut, Germany; [11] Universitätsklinikum Ulm, Ulm, Germany; [12] 1. Medizinische Klinik, Klinikum rechts der Isar, Technische Universität, Munich, Germany; [13] Universitätsklinikum Regensburg, Regensburg, Germany; [14] DZHK, Partner Site Munich Heart Alliance; [15] Munich University Clinic, Ludwig-Maximilians University, Munich, Germany

§Equal contribution first authors
*Corresponding Author

Participating Centers
Principal Investigators

Albania: Spitali Gjerman, Cardiology, Tirana; Alban Dibra (PI).

Austria: Wilhelminenspital Wien, 3. Medizinische Abteilung mit Kardiologie, Vienna; Kurt Huber (PI)
Krankenanstalt Rudolfstiftung, 2. Medizinsche Abteilung, Vienna; Franz Weidinger (PI)

Belgium: University Hospitals Leuven - Campus Gasthuisberg, Cardiovascular Diseases, Leuven; Tom Adriaenssens (PI)

China: General Hospital of Shenyang Military Region, Cardiology, Shenyang; Yaling Han (PI)

Denmark: Aarhus University Hospital Skejby, Department of Cardiology, Aarhus; Michael Maeng (PI)

Germany: Deutsches Herzzentrum München des Freistaates Bayern, Klinik an der Technischen Universität München, Klinik für Herz- und Kreislauferkrankungen, Munich; Adnan Kastrati (PI), Julinda Mehilli (PI until 10/2012)

Klinikum rechts der Isar der Technischen Universität München, 1. Medizinische Klinik und Poliklinik, Munich; Karl-Ludwig Laugwitz (PI), Josef Dirschinger (PI until 10/2010)

Universitäts-Herzzentrum Freiburg · Bad Krozingen, Klinik für Kardiologie & Angiologie, Bad Krozingen; Franz-Josef Neumann (PI)

Segeberger Kliniken GmbH, Herz-Kreislauf-Zentrum, Bad Segeberg; Gert Richardt (PI)

Helios-Klinikum Wuppertal, Klinik für Kardiologie (Medizinische Klinik 3) am Herzzentrum Wuppertal, Wuppertal; Klaus Tiroch (PI)

Krankenhaus Landshut-Achdorf, Medizinische Klinik I, Landshut; Bernhard Zrenner (PI)

Universitätsmedizin Göttingen der Georg-August-Universität, Abteilung Kardiologie und Pneumologie, Göttingen; Claudius Jacobshagen (PI); Frank Edelmann, Lars Maier; Anke Hallmann, Swetlana Hartmann, Annabelle Wolk.

Klinikum Neuperlach, Städtisches Klinikum München, Klinik für Kardiologie, Pneumologie und Internistische Intensivmedizin, Munich; Harald Mudra (PI)

MediClin Herzzentrum Lahr/Baden, Klinikum für Innere Medizin, Kardiologie, Lahr; Eberhardt von Hodenberg (PI)

Universitätsklinikum Ulm, Innere Medizin II, Ulm; Jochen Wöhrle (PI)

Universitätsklinikum Regensburg, Klinik und Poliklinik für Innere Medizin II, Regensburg; Marcus Fischer (PI), Christian Hengstenberg (PI until 12/2012)

Klinikum Garmisch-Partenkirchen, Abteilung Kardiologie, Angiologie & Pulmologie, Garmisch-Partenkirchen; Franz Dotzer (PI)

Klinikum Bogenhausen, Städtisches Klinikum München, Klinik für Kardiologie und Internistische Intensivmedizin, Munich; Martin Schmidt (PI)

Krankenhaus Barmherzige Brüder Regensburg, Medizinische Klinik III, Kardiologie, Regensburg; Peter Sick (PI)

Universitätsmedizin Rostock, Zentrum für Innere Medizin, Abteilung Kardiologie, Rostock; Christoph A. Nienaber (PI)

Charité Universitätsmedizin Berlin – Campus Benjamin Franklin, Berlin; Hans-Christian Mochmann (PI), Bernhard Witzenbichler (PI until 03/2013), Dietlind Zohlnhöfer (PI until 11/2011)

Klinikum Ingolstadt, Medizinische Klinik 1, Ingolstadt; Conrad Pfafferott (PI)

Universität Leipzig, Herzzentrum, Leipzig; Georg Fürnau (PI), Holger Thiele (PI until 01/2014)

Universitätsklinikum Schleswig-Holstein, Medizinsiche Klinik II - Campus Lübeck, Lübeck; Alexander Joost (PI), Heribert Schunkert (PI until 12/2012)

Herzzentrum Dresden, Universitätsklinik an der Technischen Universität Dresden, Klinik für Innere Medizin und Kardiologie, Dresden; Ruth H. Strasser (PI)

Universitätsklinikum Erlangen, Medizinsiche Klinik 2, Erlangen; Stephan Achenbach (PI), Werner G. Daniel (PI until 03/2013)

Klinikum der Universität München (LMU), Medizinische Klinik und Poliklinik I, Campus Großhadern, Munich; Christian Kupatt (PI)

Lukaskrankenhaus, Städtische Kliniken Neuss/Rhein, Medizinische Klinik I, Kardiologie, Neuss; Michael Haude (PI)

Ireland: University College Hospital Galway, Cardiology, Galway; Kieran Daly (PI)

Italy: Campus Bio-Medico University of Rome, Cardiology, Rome; Germano Di Sciascio (PI)

Japan: Kyoto University Hospital, Cardiology, Kyoto; Takeshi Kimura (PI)

Tokai University School of Medicine, Department of Cardiovascular Medicine, Isehara; Yuji Ikari (PI)

Netherlands: St.Antonius Ziekenhuis, Cardiologie, Nieuwegein; Jurriën M. ten Berg (PI)

Isala klinieken - locatie Weezenlanden, Cardiologie, Zwolle; Arnoud van't Hof (PI)

Maasstad Ziekenhuis Rotterdam, Cardiologie, Rotterdam; Elvin Kedhi (PI)

Catharina Hospital Eindhoven, Eindhoven, Cardiologie; Jacques J. Koolen (PI)

New Zealand: Auckland City Hospital, Cardiology, Auckland; Mark Webster (PI)

Switzerland: Universitätsklinik für Kardiologie, Schweizer Herz- und Gefäßzentrum Bern, Universitätsklinik Inselspital, Bern; Stephan Windecker (PI)

USA: University of Florida, Health Science Center, Jacksonville; Dominick Angiolillo (PI)
